# Synthesis and Properties of Waterborne Polyurethane (WBPU)/Modified Lignin Amine (MLA) Adhesive: A Promising Adhesive Material

**DOI:** 10.3390/polym8090318

**Published:** 2016-08-25

**Authors:** Mohammad Mizanur Rahman, Md. Hasan Zahir, Han Do Kim

**Affiliations:** 1 Center of Research Excellence in Corrosion, King Fahd University of Petroleum & Minerals, Dhahran 31261, Saudi Arabia; 2 Center of Research Excellence in Renewable Energy, King Fahd University of Petroleum & Minerals, Dhahran 31261, Saudi Arabia; hzahir@kfupm.edu.sa; 3 Department of Organic Material Science and Engineering, Pusan National University, Busan 608-739, Korea; kimhd@pusan.ac.kr

**Keywords:** waterborne polyurethane, adhesive, lignin

## Abstract

A series of waterborne polyurethane (WBPU)/modified lignin amine (MLA) adhesives was prepared using MLA as a chain extender by a prepolymer mixing process. A successful Mannich reaction was achieved during the synthesis of MLA by reacting lignin with bis(3-aminopropyl)amine. Higher tensile strength, Young’s modulus, and thermal stability were recorded for WBPU/MLA adhesives with higher MLA contents. The WBPU/MLA adhesive materials were used to coat polyvinyl chloride (PVC) substrates. The adhesive strength increased with increasing MLA content. More importantly, the MLA also enhanced the WBPU/MLA coating in terms of adhesive strength at moderately high temperatures as well as under natural weather exposed conditions. The adhesive strength was essentially unaffected with 6.48 mol % MLA in the WBPU/MLA coating after exposure to natural weather conditions for 180 days.

## 1. Introduction

Loosening of adhesion of a coating from a substrate over time is a common phenomenon that leads to changes in the overall coating protection. These changes will induce corrosion (for metals), fouling, or failure of the overall system, particularly in outdoor use where coatings are exposed to weathering, oxidation from the effects of UV radiation, and variation in temperature. Therefore, strong adhesive strength of a coating is crucial for long-lasting applications [1,2].

Most polymeric coatings and adhesives are generally based on fossil feedstocks. However, increasing oil prices and global warming demands a change from fossil feedstocks to renewable resources. Recently, researchers are increasingly devoting their efforts to the possible use of renewable feedstock as raw materials for the production of monomers and their polymeric coatings and adhesives. Vegetable oils and animal fats play an important role in renewable resources because of their ready availability and many versatile applications. There have been many studies of polymers based on vegetable oils, such as vinyl polymers, epoxy resins, alkyd resins, polyamides, and polyurethanes [3,4,5,6,7].

Polyurethane (PU) is a widely used material in coating and adhesive industry because it exhibits superior adhesive strength, mechanical strength, and UV resistance [8,9,10,11,12,13,14,15,16,17,18]. Earlier interest was in solvent-based PU, in which different organic solvents were used to synthesize PU. However, the rapid rise of solvent price and increased environmental concerns are pressuring the scientists for the use of water as the main solvent in PU applications. In this regard, waterborne polyurethane (WBPU) can be a good choice due to the use of water as a main solvent during their synthesis. There are different monomers to prepare WBPU dispersions. Widely used monomers such as polyols, diisocyanates, hydrophilic agents with pendant acid group, neutralizing agents, and chain extenders are mostly petroleum-based materials [16,17,18]. Recently, plant-resourced monomers have been successfully used to synthesize WBPU [19]. Different polyols were considered in this respect. Chain extender is another important monomer for synthesizing WBPU. The mechanical strength and adhesive strength highly depends on the chain extenders and their contents. Ethylenediamine (EDA) is a widely used chain extender in WBPU, and the properties change dramatically when using different EDA content [17]. Unfortunately, EDA is harmful for human health, and thus needs to be replaced by natural resources.

The properties can also be further improved by using proper renewable resource fillers, such as tannins and lignins [20,21,22]. Lignin, a promising renewable resource filler for PU, is composed of phenolic units and is the second most abundant plant biomass component after cellulose. In industry, lignins are typically obtained as byproducts or waste from papermaking and emerging cellulosic ethanol production. The rigid structure of lignin allows for its utilization as a low-cost reinforcement filler. The properties of PU, epoxy, and acrylate have been improved by using proper lignin contents. A homogeneous lignin–PU material exhibit enhanced mechanical properties, thermal stability, and UV protection [23].

Unfortunately, the use of lignin in WBPU coating and adhesive materials is very scarce. One of the challenges of WBPU/lignin dispersion is its stability and shelf life. To improve the stability of WBPU/lignin dispersion, J. Liu et al. modified lignin using diethylenetriamine through a Mannich reaction [19]. Superior mechanical and anti-aging properties were recorded following the above technique. Typically, an alkyl group in the coating can improve the hydrophobicity and thermal resistance [24]. Although there have been some attempts to use larger alkyl chains in different ways, the larger alkyl groups lead to WBPU dispersion challenges. Increasing the dimethylolpropionic acid (DMPA) content is one way to use larger alkyl chains in WBPU. Another way to modify the monomer is by reacting the targeted alkyl group. In this study, the lignin was modified by using bis(3-aminopropyl)amine (BAPA) to make modified lignin amine (MLA) as a chain extender. Three different chain extenders (single EDA, single MLA, and mixture of EDA and MLA) were used in dispersions and their performances were compared. The MLA was used up to 6.48 mol %. The dispersions were used to coat a polyvinyl chloride (PVC) surface, and the adhesive strength after exposure for a defined interval to both a hot chamber and natural weather conditions (separately) was evaluated. The outside exposure time was 180 days. Here, the PVC was considered a base specimen to avoid corrosion during the exposure time. The thermal properties of the coatings were analyzed by thermogravimetric analysis (TGA) and differential scanning calorimetry (DSC). The mechanical properties were evaluated through tensile tests. X-ray photoelectron spectroscopy (XPS) analysis was conducted for analysis of degraded coatings.

## 2. Experimental

### 2.1. Materials

Poly(tetramethyleneoxide glycol, PTMG, *M*_n_ = 2000, Sigma Aldrich, Saint Louis, MO, USA) was vacuum dried at 90 °C for three hours prior to use. Dimethylolpropionic acid (DMPA, Sigma Aldrich), triethylamine (TEA, Sigma Aldrich), *N*-methyl-2-pyrrolidone (NMP, Sigma Aldrich), 4,4-dicyclohexylmethanediisocyanate (H_12_MDI, Sigma Aldrich) and EDA (Sigma Aldrich) were used after dehydration with 4 Å molecular sieves for seven days. Dibutyltindilaurate (DBTDL, Sigma Aldrich), lignin (Sigma Aldrich) and BAPA (Sigma Aldrich) were used as received.

### 2.2. Preparation of Lignin Amine

The lignin amine was synthesized according to the modified method of Liu et al. [19]. Lignin (5 g) and BAPA (7.5 g) were mixed in 50 mL of distilled water. The pH of the mixture was maintained at 10–10.5 with 0.1 M NaOH and HCl solutions. The mixture was added to a three-necked flask, to which 5 g of formaldehyde solution (37 wt %) was added stepwise while stirring at room temperature. Next, the mixture was heated to 50 °C for 6 h. Afterward, the solution was added to approximately 350 mL of isopropyl alcohol to precipitate the lignin amine. The resulting light brown precipitate was recovered using suction filtration, washed with isopropyl alcohol four times, and then dried in a vacuum oven at 50 °C.

### 2.3. Preparation of WBPU Dispersion

This dispersion was prepared by a prepolymer process following our previous report [18]. The prepolymer was prepared by charging polyol, 2,2-dimethylol propionic acid, and H_12_MDI for 3 h at 85 °C. The prepolymer was neutralized using proper amount of TEA. Water was added in the dispersion step, whereas EDA was added in the chain extension step.

### 2.4. Preparation of WBPU/MLA Dispersions

The sample designation and composition of the WBPU/MLA dispersions are summarized in Table 1. WBPU/MLA dispersions were prepared using the prepolymer process. Initially, MLA was mixed with H_12_MDI using sonication. This mixture was added to another vessel containing a mixture of PTMG/DMPA/NMP/DBTDL solution to make isocyanate group (NCO)-terminated prepolymer, followed by neutralization with TEA. A calculated amount of water (30 wt %) was added to the mixture to disperse the prepolymer in water. In the last step, EDA/MLA was added to the mixture. The residual NCO group reacted with the amine group and terminated the reaction, which was confirmed by the disappearance of the NCO group by Fourier-transform infrared (FTIR) analysis.

### 2.5. Preparation of the Films

The films were prepared by pouring aqueous dispersions (10 g) onto a Teflon disc (diameter of 7 cm), followed by drying under ambient conditions for 48 h. The films (typically ~0.5 mm thick) were also dried at 60 °C for 6 h and vacuum dried at 80 °C for 6 h. The vacuum-dried films were stored in a desiccator at room temperature.

### 2.6. Mounting of Coatings onto the PVC Solid Supports

The dispersion was mounted onto flat PVC solid supports with an applicator. The coated samples (200 μm under wet conditions) were dried under ambient conditions for 48 h.

### 2.7. Curing of the Coatings

Both curing temperature and time are important factors in the adhesive strength of the coatings. To avoid any unwanted reactions, the optimum curing time and temperature were determined to be 5 min and 100 °C, respectively.

### 2.8. Characterization

FTIR spectroscopy (Impact 400D, Nicolet, Madison, WI, USA) was used to identify the composite structure. The dispersion was coated on a thalliumbromide/thalliumiodide crystal surface as a thin liquid film and dried for analysis. For each sample, 32 scans at a resolution of 4 cm^−1^ were collected in transmittance mode.

The mechanical properties (tensile strength, Young’s modulus, and elongation at break) were measured at room temperature using a United Data Systems tension meter (SSTM-1, United Data Systems, Tokyo, Japan) according to the ASTM D 638 specifications. A crosshead speed of 50 mm/min was used throughout these studies. The values quoted are the means of five measurements.

Thermal gravimetric analysis was performed in a Pyris 6 TGA (Perkin Elmer, Shelton, CT, USA). Five milligrams of film sample was placed in a platinum pan and heated from 30 to 500 °C in a nitrogen atmosphere, at a heating rate of 10 °C/min.

The thermal behavior of the WBPU film was analyzed by DSC (TA, New Castle, DE, USA). WBPU film (3–4 mg) was placed in an aluminum pan and the experiment was carried out under nitrogen gas atmosphere at a heating rate of 10 °C/min.

The adhesive properties (peel resistance of the adhesive, i.e., T-peel strength) were measured using a universal testing machine according to ASTM D 1876-01. A peel rate of 100 mm/min was used. The values quoted are the means of five measurements.

The polymer surface was analyzed by XPS (ESCA 250 XPS, Thermo Scientific, East Grinstead, UK).

## 3. Results and Discussion

**Figure 1:**
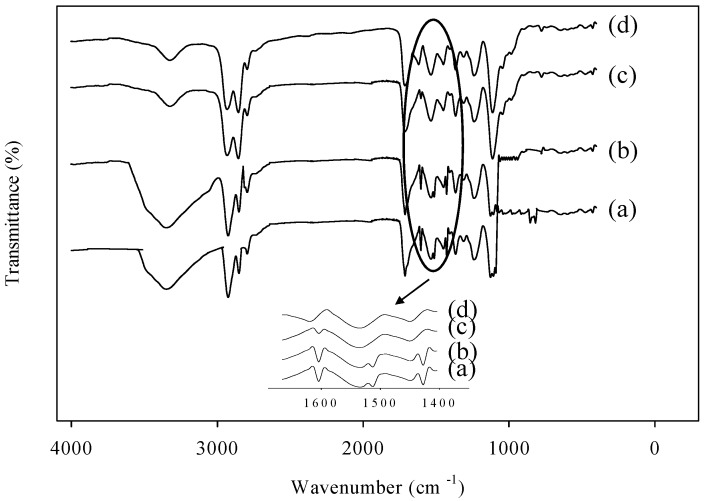
FTIR spectra of (**a**) lignin, (**b**) modified lignin amine (MLA), (**c**) WBPU/MLA-3 (lignin 4.63 mol %) and (**d**) WBPU/MLA-0 (without lignin).

The lignin was modified as in previous report [19]. Figure 1 shows that the FTIR peaks corresponding to the pristine lignin, MLA, and WBPU/MLA, respectively. Pristine lignin shows a broad band at 3500–3000 cm^−1^, which is attributed to the hydroxyl groups. The bands centered approximately 2925 and 2843 cm^−1^ arise from CH_2_ stretching. In the carbonyl region, medium bands observed near 1710 cm^−1^ are associated with conjugated carbonyl stretching and the three bands at 1606, 1515, and 1425 cm^−1^ are characteristic of aromatic rings from aromatic skeleton vibrations. The FTIR spectrum of MLA showed that the relative intensity of the broad peak at 3500–3000 cm^−1^ increased, and this was attributed to the introduction of amino groups. Besides, the greatly weakened band of aromatic stretching vibrations at 817 and 856 cm^−1^ confirmed that the benzene ring of lignin was functionalized via the reaction [25].

A minimum DMPA content is required for the formation of stable conventional WBPU dispersions. A higher DMPA content is typically needed to disperse cross-linker or additional nanoparticles. However, the dispersion with MLA was stable as with pristine WBPU with the same DMPA content. Different MLA contents were used in the WBPU/MLA dispersions, which were stable like their respective pristine WBPU dispersions. The synthesis of the WBPU/MLA dispersions was confirmed using FTIR analysis (see Figure 1). The absence of a peak within the 2000–2300 cm^−1^ range indicates that all of the isocyanate groups reacted [18,19]. All of the spectra exhibit the characteristic bands at 3150–3600, 2800–3000, 2795, 1600–1760, and 1109 cm^−1^, which correspond to NH, CH, O–CH_2_, C=O, and C–O–C stretching in the ether group, respectively [17,18]. These peaks are identical for PU and confirm the preparation of the WBPU/MLA dispersions.

The mechanical properties of the WBPU/MLA films were evaluated using tensile tests. The corresponding Young’s modulus, tensile strength, and elongation at break (%) of the films are summarized in Table 2. The mechanical properties of the films varied slightly according to the low MLA content (1.39 mol %). Significant mechanical changes were recorded when the MLA content exceeded 4.63 mol %. The tensile strength and Young’s modulus of the WBPU/MLA films increased with increasing MLA content. Maximum tensile strength and Young’s modulus were observed at an MLA content of 6.48 mol %. The tensile strength and Young’s modulus increased 38% and 240%, respectively, using MLA (sample WBPU/MLA-4) comparing to those without MLA (sample WBPU/MLA-0). This observation can be ascribed to the insertion of an ideal amount of the MLA into the matrices, resulting in the formation of strong interfacial interactions between the flat MLA and the polymer chains. This restricts the mobility of the polymer chains and creates a compact structure, which requires high energy input for elongation, which is reflected in the higher tensile strength and Young’s modulus of the films.

**Figure 2:**
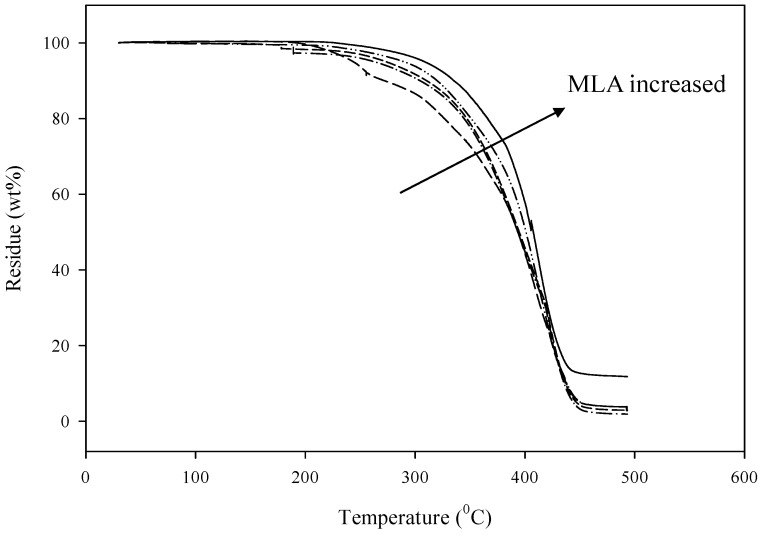
Thermogravimetric analysis (TGA) of WBPU/MLA films with different MLA content.

Thermal stability of polymers is typically determined using TGA analysis (see Figure 2). TGA curves indicate the weight loss (%) of materials with respect to the temperature of thermal degradation [18]. The thermal degradation of pristine WBPU can be divided into main two stages. The first stage at 250 °C corresponds to the urethane group, whereas the second stage at 390 °C mainly accounts for the urea group [18]. Using different chain extenders did not alter the stages; degradation for all films was in two stages. However, the addition of MLA increased the thermal stability in both stages. This thermal stability continued to increase with increasing MLA content. The stiff aromatic ring, as well as direct linkage of the MLA group into the PU chain, enhanced the overall thermal stability.

**Figure 3:**
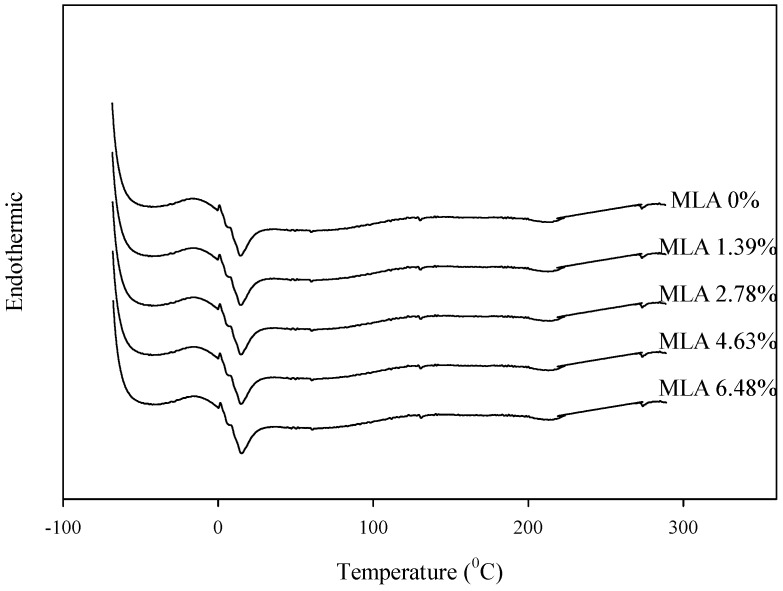
Differential scanning calorimetry (DSC) of WBPU/MLA films with different MLA content.

The thermal properties of the WBPU/MLA films were studied using DSC, the results of which are summarized in Figure 3. All of the samples containing MLA were shown to have almost the same melting peaks for the soft segment (*T*_m_ SS) at approximately 19 °C (see Figure 3). The heat of fusion (18.5 mJ/mg) remained nearly unchanged for different MLA contents. The melting temperature of the hard segment was not observed, indicating the amorphous structure of the hard segment regions of the WBPU/MLA coatings. One possible explanation for this observation is that the MLA disrupts the hard segment crystallinity and that the peak for the hard segment was not observed.

**Figure 4:**
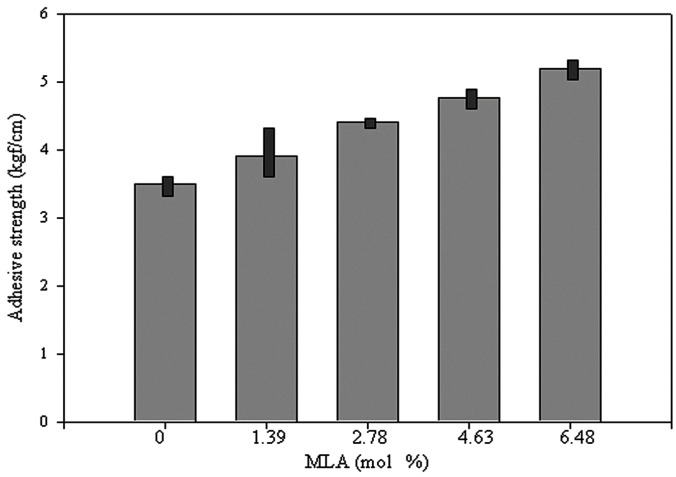
Effect of MLA content on adhesive strength of coatings at ambient condition.

**Figure 5:**
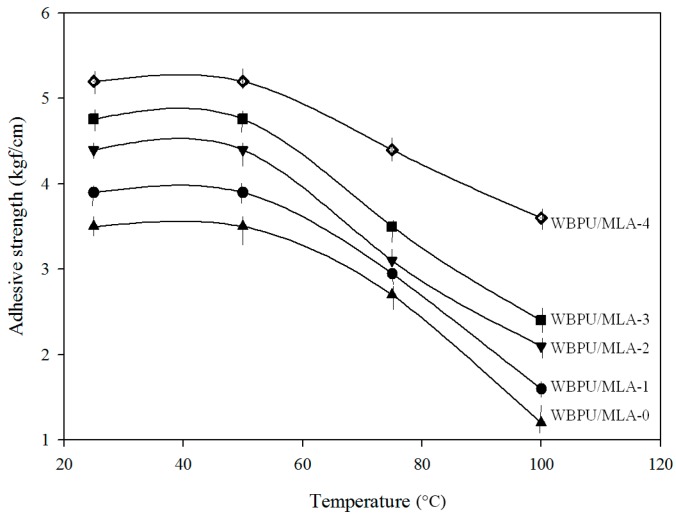
Adhesive strength of WBPU/MLA coatings at different temperature.

The coating properties were evaluated by adhesive strength and mechanical damage. Visually, there was no mechanical damage to the coatings before exposure to a hot chamber or to outside conditions. The adhesive strength gradually increased with increasing MLA content before exposure, which may be attributed to the higher mechanical strength with increasing MLA content. The aromatic planar lignin made the coating very stiff and improved the adhesive strength. The mean adhesive strength value is shown in Figure 4. The adhesive strength changed after exposure to different defined temperatures (see Figure 5). For all samples, the adhesive strength decreased with increasing temperature. However, the decreased rate of the adhesive strength was different for all coatings; the rate decreased with increasing MLA content. This implies that the degradation rate might be decreased with increasing MLA content. The coating with 6.48 mol % MLA exhibited the lowest decreased rate of approximately 30% at 100 °C, whereas, the coating without MLA (only EDA) exhibited the highest decreased rate of 67% at 100 °C. These result imply a moderately resistant adhesion of WBPU/MLA with 6.48 mol % MLA at moderately high temperatures. A good combination of high thermal stability and mechanical strength of the WBPU/MLA coatings made the difference in adhesive strength compared to WBPU without lignin at moderately high temperatures.

**Figure 6:**
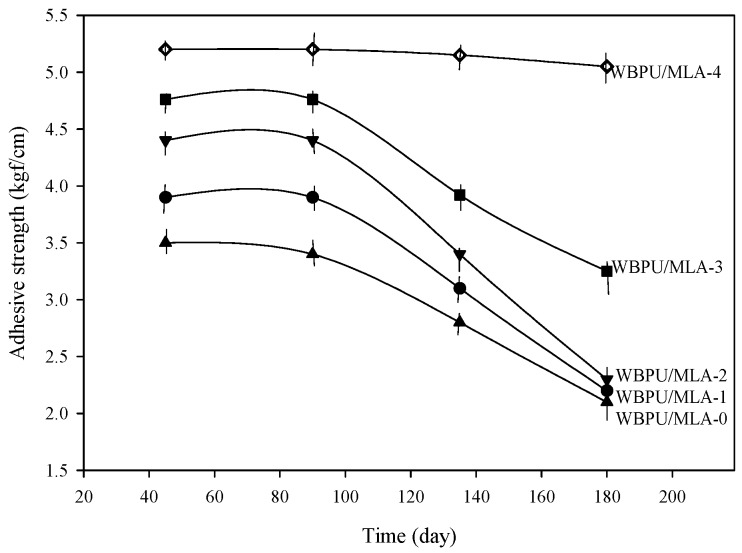
Adhesive strength of WBPU/MLA coatings at different exposure time.

**Figure 7:**
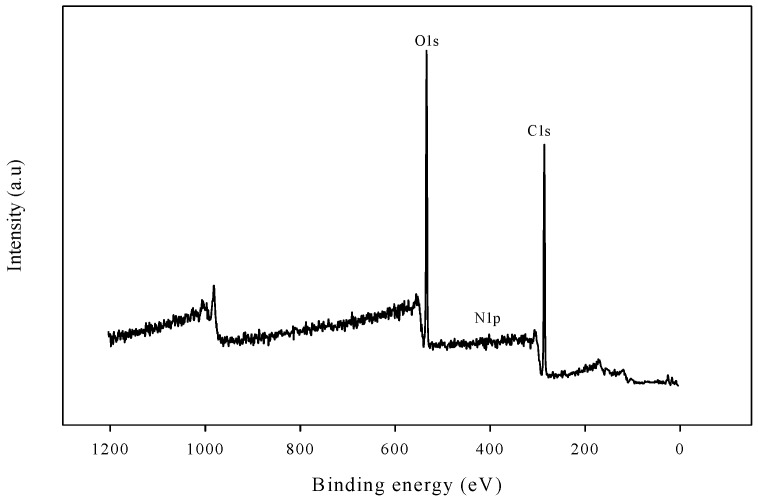
X-ray photoelectron spectroscopy (XPS) spectra of WBPU/MLA-4 coating.

**Figure 8:**
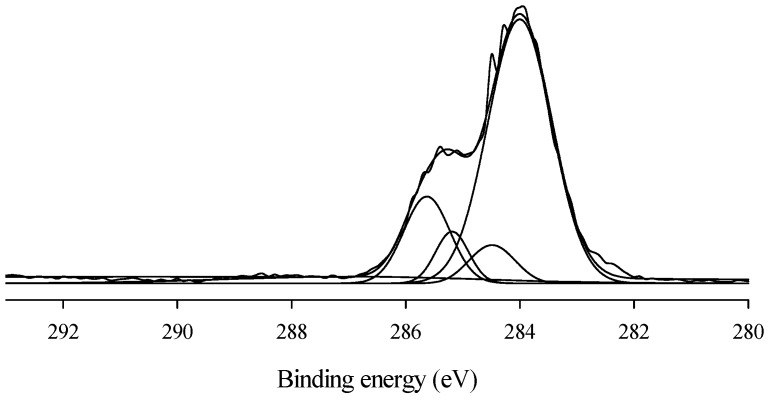
Deconvulated spectra of WBPU/MLA-4 coating.

**Figure 9:**
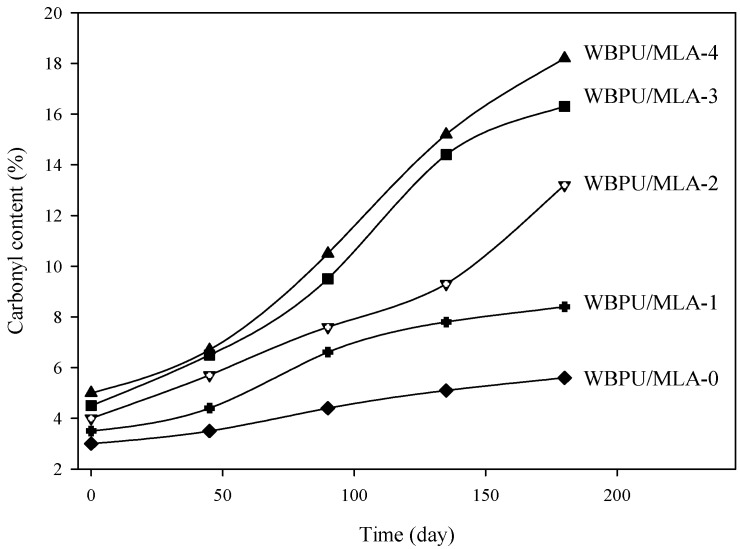
Carbonyl content of exposed WBPU/MLA coatings.

The adhesive strength was also evaluated after exposure of coating to natural weather conditions for 180 days. The adhesive strength of exposed coatings is summarized in Figure 6. It was recorded that the adhesive strength decreased with increasing exposure time. However, the adhesive strength was less affected in the WBPU/MLA coatings. The adhesive strength decreased only 3% after 180 days of exposure of the WBPU/MLA (6.48 mol %) coatings. The adhesive strength of the exposed coating might be highly dependent on the coating degradation rate. A degraded coating has less resistivity of barrier, allowing for more facile passing of water, air, and other unwanted dusts, which help to decrease the bond strength of the coating. In this case, the MLA acted as a UV absorber and protected the coating from degradation. Visually, the change in coatings was very minor. However, a large degradation was recorded through the XPS technique. The peaks for certain atoms were found to be nearly identical in all exposed coatings (see Figure 7). The peaks at 531, 285, and 400 eV attributed to oxygen (1s), carbon (1s), and nitrogen (1p), respectively [24], were observed in the survey spectra for all the exposed coatings. Because the oxygen (1s), carbon (1s), and nitrogen (1p) atoms have different binding energies, their peaks are observed at different values. However, for the carbon atoms in different environments with different functional groups, the C1s binding energies of these functional groups are very close to each other and their original functional groups can be determined using curve fitting analysis. The C1s peaks are classified into five groups: C=O carbon atom appearing at 289.7–285.8 eV, C–C or C–H appearing at 282.0–285.9 eV, C–O appearing at 284.1–286.1 eV, C–N appearing at 284.5–287.1 eV, and aromatic ring appearing at 283.20–285.68 eV (see Figure 8) [24,26]. With the inclusion of MLA, the area at 289.7–285.8 eV (for the carbonyl groups) decreased (not shown). As reported elsewhere, the carbonyl contribution is attributed to the urethane/urea groups of WBPU. A smaller carbonyl area implies a lower content of urethane/urea groups on the surface. The lowest carbonyl area was determined for the highest MLA content of 6.48 mol % (not shown), implying that the maximum amount of buried urethane/urea groups by MLA on the surface was found at this composition. Usually the coatings exposed to open air atmosphere are susceptible to degradation, leading to eventual loss of adhesion. Carbon dioxide and carbon monoxide are two prominent degrading products, which can be detected by the presence of carbonyl groups through XPS analysis. The increased carbonyl content of the exposed coatings implies the degradation of the coatings and eventually affects the adhesive strength of the coatings. The carbonyl contents of all the exposed coatings are shown in Figure 9. The carbonyl content changed significantly for the pristine WBPU exposed coating, which further increased with time. This degradation resulted in the facile passing of water/air through the coating into the PVC and directly impacted the adhesive strength of the coating. Very low adhesive strength was observed in this case. However, the carbonyl content was different when 4.63 mol % or higher MLA content was used in the coatings. The carbonyl content was observed to be lowest for the WBPU/MLA coating with 6.48 mol % MLA (see Figure 9). This result can be ascribed to the lowest chain scission of urethane/urea groups in the presence of UV (sunlight). Herein, the MLA acted as a UV absorber, which protected the chain scission of urethane/urea groups, eventually decreasing the degradation of the coating. Hence, the adhesive strength was nearly unaffected, especially at 6.48 mol % MLA content.

## 4. Conclusions

Naturally resourced lignin was modified through Mannich reaction using bis(3-aminopropyl)amine to make modified lignin amine (MLA) which was used as a chain extender in WBPU/MLA dispersions. The thermal stability, UV degradation resistivity, and adhesive strength all increased using MLA as a chain extender. Regarding the adhesive strength at moderately high temperatures and under exposed conditions, the WBPU coating with EDA showed muchless resistance, whereas, the WBPUcoating with MLA showed high resistance. The adhesive strength of the WBPU/MLA coating (using 6.48 mol % MLA, without EDA) decreased 30% and 3% at 80 °C and under exposed conditions, respectively. Considering the good adhesive strength at different conditions, the MLA can be considered an alternative to the current toxic EDA in WBPU materials, which will create a new branch of application of green products.

## Figures and Tables

**Table 1:** Composition of waterborne polyurethane (WBPU)/modified lignin amine (MLA) dispersions. PTMG, poly(tetramethyleneoxide glycol); H_12_MDI, 4,4-dicyclohexylmethane diisocyanate; DMPA, dimethylolpropionic acid; TEA, triethylamine; EDA, ethylenediamine.

Sample	Mole ratio					MLA (mol %)
	PTMG	H_12_MDI	DMPA	TEA	EDA/MLA	
WBPU/MLA-0	0.040	0.108	0.054	0.054	0.014/0	0
WBPU/MLA-1	0.040	0.108	0.054	0.054	0.011/0.003	1.39
WBPU/MLA-2	0.040	0.108	0.054	0.054	0.008/0.006	2.78
WBPU/MLA-3	0.040	0.108	0.054	0.054	0.004/0.010	4.63
WBPU/MLA-4	0.040	0.108	0.054	0.054	0/0.014	6.48

**Table 2:** Mechanical properties of WBPU/MLA films.

Sample	Tensile strength (MPa)	Young’s modulus (MPa)	Elongation at break (%)
WBPU/MLA-0	32 ± 0.2	5 ± 0.3	950 ± 0.4
WBPU/MLA-1	34 ± 0.3	6 ± 0.2	930 ± 0.3
WBPU/MLA-2	36 ± 0.2	8 ± 0.1	901 ± 0.4
WBPU/MLA-3	39 ± 0.2	11 ± 0.1	840 ± 0.6
WBPU/MLA-4	44 ± 0.1	17 ± 0.1	804 ± 0.9
